# Efficacy of Influenza Vaccination in Patients with Cirrhosis and Inactive Carriers of Hepatitis B Virus Infection

**Published:** 2012-10-30

**Authors:** B Sayyad, S M Alavian, F Najafi, T Mokhtari Azad, M H Ari Tabarestani, M Shirvani, B Behnava, M Afshrian, S Vaziri, A R Janbakhsh, F Mansouri, Sh Kaviani

**Affiliations:** 1 Kermanshah Liver Diseases and Hepatitis Research Center, Kermanshah University of medical Sciences, Kermanshah, Iran; 2 Baqyatallah Research Center for Gastroenterology and Liver Disease, Tehran, Iran; 3 Kermanshah Health Research Center, Kermanshah University of medical Sciences, Kermanshah, Iran; 4 Department of Virology, School of Public Health, Tehran University of Medical Sciences, Tehran, Iran

**Keywords:** Influenza, Vaccination, Cirrhosis, Hepatitis B

## Abstract

**Background:**

Influenza can causes morbidity and mortality that are greatly enhanced in patients with underlying chronic diseases such as Cirrhotic patients. This study was performed to assess the immunogenicity of Influenza vaccination in patients with cirrhosis and inactive carriers of Hepatitis B virus infection.

**Methods:**

In this clinical study 93 enrolled subjects divided into 3 groups: Cirrhotic (N=28), Inactive carriers of Hepatitis B (N=31) and subjects (N=34). All the participants were vaccinated by Influenza vaccine (Influvac®). Serum samples were taken before and 4 weeks after vaccination and the Humoral Immunogenicity was assessed by the Hemagglutination Inhibition (HI) test.

**Results:**

Four weeks after vaccination, seroconversion rates of vaccine strains ranged between 71.4% and 100% in Group 1, 70.6% and 94.1% in Group 2, and 58.1% and 80.7% in Group 3. No significant differences were seen in the rates of Seroconversion and antibody Geometric Mean Titers (GMTs) against Influenza A (H1N1 and H3N2) vaccine components in the three groups (P>0.05).The rates of Seroconversion and antibody GMTs against Influenza B vaccine component were significantly higher in Cirrhotic and inactive carriers of Hepatitis B than healthy subjects (P<0.005). No significant (P>0.05) differences in the rates of Seroprotection were observed within the three groups. Antibody GMTs against all three strains of Influenza vaccine increased significantly (P<0.001) after vaccination in three groups.

**Conclusion:**

Influenza vaccination is effective in Cirrhotic patients and inactive carriers of Hepatitis B as well as healthy individuals. It means that vaccination should be considered in such patients in order to reduce the morbidity and mortality of Influenza.

## Introduction

Chronic Hepatitis B Virus (HBV) infection is a global health problem associated with diverse human liver disorders ranging from chronic Hepatitis to cirrhosis and Hepato Cellular Carcinoma (HCC).(1) Iran is low to intermediate prevalence of HBV and vaccination against HBV has decreased prevalence(2,4), but it remains the main cause of chronic liver disease in Iran.(5,6)

Influenza is an acute, febrile and, usually, self-limited illness which is induced mainly by types A and B of Influenza virus leading to outbursts of variable extent in Autumn and Winter such as H1N1 Influenza pandemics in 2009 and crowded population such as Influenza outbreaks in Hajj. The most common manifestations of the illness are fever, lassitude and cough. Two main features of Influenza are epidemics and high mortality which are mostly due to viral pneumonitis, bacterial pneumonia and ARDS. Other complications include Myocarditis, Pericarditis, Myositis, Guillain-Barre syndrome, Transverse Myelitis, and Encephalitis.(7,9)

Complicated Influenza and its mortality often occurs in individuals with underlying problems. Senility, chronic renal, hepatic and cardiopulmonary diseases, hematologic disturbances, metabolic diseases such as diabetes mellitus, immune defects, HIV infection, and pregnancy are the underlying problems leading to more frequent complications and high mortality in Influenza- afflicted patients.(7)

Patients with Chronic liver disease are vulnerable to severe complications of Influenza through numerous Pathophysiological mechanisms of which one can refer to Reticuloendothelial dysfunctions, defects in the production of complement components, Neutrophil and T & B Lymphocytes dysfunctions, abnormal production of Immunoglobulin and, also, Macrophage dysfunctions. (10)

On the other hand, Influenza may systematically affects on the patients and leads to aggravation of chronic liver disease. Among which one can mentions direct damages to Hepatocytes by Influenza virus, direct damages induced by circulating Cytokines in the course of Influenza and, also, seriousness of the underlying Pulmonary disturbances in Cirrhotic patients.(11) Indeed, in patients with chronic liver disease, Influenza and the consequent secondary bacterial infections may lead to an increased load of severe complications as hospital admissions and mortality.(12) However, if the annual Influenza vaccination be effective, so it can be a logical and cost-effective way of reducing complications and mortality of Influenza in vulnerable patients who are afflicted with chronic liver disease and cirrhosis. Although some studies indicated the efficacy of Influenza vaccination in Cirrhotic patients(12,15), but American Centre for Disease Control (ACDC) has not classified these patients as part of the target population of annual Influenza vaccination.(16)

The present study investigated the efficacy of Influenza vaccination in Cirrhotic patients, as a vulnerable group for developing complications, and inactive carriers of Hepatitis B, which Influenza may directly or indirectly leads to liver damage, as compared with healthy subjects.

## Materials and Methods

### Individuals under Investigation

In this clinical study, from 23 Oct, 2009 to 21 Apr, 2010, only 93 of 114 patients who participated in the study to the end. These participants were examined in three groups: Cirrhotic patients (28), inactive carriers of Hepatitis B (31 subjects) and health cases (34 subjects). The healthy group was negative for HBsAg, HCV Ab, and HIV Ab and had no specific illness in their history. All members of the inactive carriers of Hepatitis B group were positive for HBsAg, negative for HBeAg, and patients who was positive for HBe Ab, had normal ALT levels (less than 30 u/l for men and less than 19 u/l for women), and their Hepatitis B viral loads were less than 10,000 cop/ml (By Amplicor test version 2 Rouch). Cirrhotic cases were diagnosed by liver histology of fibrosis and nodular regenerative formation. We took history of previous Influenza vaccination status but did not collect any information about potential exposure or symptoms of Influenza in same or previous season.

The Ethical Committee of Baqiyatallah University of Medical Sciences and Kermanshah University of Medical Sciences has approved the study (Ethical code: 48175/420/7). The IRCT code is 138812103456N1. After taking informed consent, a questionnaire which was contained required data for the investigation was filled by patients. The main data related to patients has been summarized in Table 1. From each patient under investigation, 5 cc venous blood was taken, the serum of which was kept at a temperature of -20 degree Celsius after being separated. The vaccine in use, trivalent Influenza vaccine (Solvay, Netherlands, Influvac®) was related to years 2009/2010. This vaccine contained 15µg Hemagglutinin (HA) related to each of the following strains:

**Table 1:** Seroprotective Level and Antibody GMTs before Immunization in the three Groups of Study Participants

	Group 1, Cirrhotic Patient (N=28)	Group 2, Inactive Carriers of Hepatitis B (N=31)	Group 3, Healthy Individuals (N=34)	*P Value*
Seroprotective level(%)				
A/H1N1b	67.9	52.9	64.5	0.44
A/H3N2c	71.4	52.9	67.7	0.27
Bd	64.3	20.6	48.4	0.002
GMTs				
A/H1N1b	31.2 (2.0)	24.1 (2.4)	32.7 (2.6)	0.30
A/H3N2c	36.6 (2.4)	24.5 (2.3)	30.0 (2.6)	0.28
Bd	27.1 (2.1)	14.9 (2.1)	23.3 (2.2)	0.006

A/Brisbane/59/2007 (H1N1)-like strain (A/Brisbane/59/2007 IVR-148 reass), A/Brisbane/10/2007 (H3N2)-like strain (A/Uruguay/716/2007 NYMC X-175C reass) and B/Brisbane/60/2008 (H3N2)-like strain (B/Brisbane/60/2008).

The vaccine was injected deep subcutaneously in the deltoid area.

### Immunogenicity Assay

The blood samples collected pre- and post-vaccination were sent to National Influenza Center in School of Public Health, Tehran University of Medical Sciences. 

The humoral immune response was investigated by use of Hemagglutination Inhibition (HI) test and standard micro-titer assay. 

This test measures the titer of serum antibody against Hemagglutinin antigens of A/H3N2 and B A/H1N1 vaccine strains. In this test, the highest serum dilution in which agglutination is inhibited is considered as the HI antibody titer. The laboratory was blinded to enrolled persons situation.

Immunogenicity is assayed in terms of the following definitions:

A) Seroprotection: the existence or production of antibody with titers more than or equal to 1: 40 which may induce protection against Influenza.

B) Seroconversion: Increase of the antibody titers from negative (less than 1:40) to positive (more than or equal to 1:40) or 4 times or more increase of the antibody following vaccination.

C) Geometric Mean Titers (GMTs): the significant increase of the mean geometric titer of the antibody following vaccination. In this study, for calculating antibody GMTs, 1:5 titers have been considered as the basis of the minimum titer.

### Statistical Analysis

In order to compare the baseline characteristics and demographic data, we used chi-square (for categorical variables) and one-way ANOVA for continuous variables. By using pair T-test, we compared the GMT of Ab production between baseline and after vaccination in each group. Furthermore, we compared the Seroconversion and Seroprotection between the three groups by chi-square test. Finally, we compared the GMT of Ab production between the three groups who was using one-way ANOVA. We used STATA 8 and the level of significance was assumed <0.05.

## Result

The main characteristics of the study’s participants’ are summarized in Table 1. As it is shown, no significant difference was observed between the three groups in sex and BMI, but mean age was significantly higher in cCirrhotic group, and also these patients had not any history of vaccination against Influenza.

Before vaccination, Sero-protective levels and antibody GMTs against H1N1and H3N2 strains had not significant difference in the three groups, but Cirrhotic patients had higher Sero-protective and GMTs antibody against B strain of Influenza virus (P=0.002 and P=0.006, respectively) (Table 1).

As it is shown in Table 3, post-vaccination Seroprotection rate in the first group (cirrhotic patients) was 100% for all strains, in the second group inactive carriers of Hepatitis B was 100% for the B and H1N1 strains and 91.20% for H3N2, and in the third group (healthy subjects) it ranged from 93.60% to 96.80 %. No significant statistical difference was observed between the three groups in Seroprotection rate.

Seroconversion rate following vaccination for vaccine strains was as follows: 71.4% to 100% in the first group – (Cirrhotic patients), 70.6% to 94.1% in the second group (inactive carriers of Hepatitis B), and 58.1% to 80.7% in the third group (healthy subjects) (Table 2).

**Table 2:** Seroprotection, Seroconversion and Antibody GMTs before and after Immunization in the three Groups of Study Participants

	*Group *1, Cirrhotic Patient (N=28)				*Group *2, Inactive Carriers of Hepatitis B (N=31)				*Group *3, Healthy Individuals (N=34)				*P*^a^
**Seroprotection (%)**	**Pre-Vaccination**		**Post-Vaccination**		**Pre-Vaccination**		**Post-Vaccination**		**Pre-Vaccination**		**Post-Vaccination**		
**A/H1N1b**	67.9		100.0		52.9		100.0		64.5		96.8		0.36
**A/H3N2c**	71.4		100.0		52.9		91.2		67.7		96.8		0.22
**Bd**	64.3		100.0		20.6		100.0		48.4		93.6		0.13
**Seroconversion(%)**	**Post-Vaccination**				**Post-Vaccination**				**Post-Vaccination**				
**A/H1N1b**	92.8				91.2				80.7				0.28
**A/H3N2c**	71.4				70.6				58.1				0.46
**Bd**	100				94.1				74.2				0.003
**Antibody GMTs**	**Pre-Vaccination**	**Post-Vaccination**		**P**^b^	**Pre-Vaccination**	**Post-Vaccination**		**P**^b^	**Pre-Vaccination**	**Post-Vaccination**		**P**^b^	
**A/H1N1b**	31.2 (2.0)	170.7 (1.6)		< 0.001	24.1 (2.4)	177.7 (1.8)		<0.001	32.7 (2.6)	164.0 (1.9)		<0.001	0.67
**A/H3N2c**	36.6 (2.4)	121.5 (1.9)		< 0.001	24.5 (2.3)	111.1 (2.4)		< 0.001	30.0 (2.6)	109.9 (2.1)		< 0.001	0.67
**Bd**	27.1 (2.1)	210.6 (1.7)		< 0.001	14.9 (2.1)	134.3 (2.0)		< 0.001	23.3 (2.2)	109.9 (2.3)		< 0.001	0.002

^a^P-value: Inter Groups

^b^P-value: Before &amp; after in each group

**Table 3:** Seroprotection, Seroconversion and Antibody GMTs before and after Immunization in the three Groups of Study Participants.

	*Group *1, Cirrhotic Patient (N=28)					*Group *2, Inactive Carriers of Hepatitis B (N=31)				*Group *3, Healthy Individuals (N=34)				** P^a^
Seroprotection)	**Pre-Vaccination**		**Post-Vaccination**			**Pre-Vaccination**		**Post-Vaccination**		**Pre-Vaccination**		**Post-Vaccination**		
A/H1N1b	67.9		100.0			52.9		100.0		64.5		96.8		0.36
A/H3N2c	71.4		100.0			52.9		91.2		67.7		96.8		0.22
Bd	64.3		100.0			20.6		100.0		48.4		93.6		0.13
Seroconversion	**Post-Vaccination**					**Post-Vaccination**				**Post-Vaccination**				
A/H1N1b	92.8					91.2				80.7				0.28
A/H3N2c	71.4					70.6				58.1				0.46
Bd	100					94.1				74.2				0.003
Antibody GMTs	**Pre-Vaccination**	**Post-Vaccination**		**P**^b^	**Pre-Vaccination**		**Post-Vaccination**		**P**^b^	**Pre-Vaccination**	**Post-Vaccination**		**P**^b^	
A/H1N1b	31.2 (2.0)	170.7 (1.6)		< 0.001	24.1 (2.4)		177.7 (1.8)		<0.001	32.7 (2.6)	164.0 (1.9)		<0.001	0.67
A/H3N2c	36.6 (2.4)	121.5 (1.9)		< 0.001	24.5 (2.3)		111.1 (2.4)		< 0.001	30.0 (2.6)	109.9 (2.1)		< 0.001	0.67
Bd	27.1 (2.1)	210.6 (1.7)		< 0.001	14.9 (2.1)		134.3 (2.0)		< 0.001	23.3 (2.2)	109.9 (2.3)		< 0.001	0.002

^a^P-valuea: Inter Groups

^b^P-valueb: Before &amp; after in each group

## Discussion

Seroconversion for H1N1and H3N2components of vaccine in all three groups showed no significant statistical difference. However, Seroconversion for B component of Influenza vaccine showed a significant statistical difference between the three under investigation groups, as Cirrhotic patients who showed more Seroconversion in compare with the inactive carriers of Hepatitis B and the healthy subjects (Table 2).

The antibody GMTs against all three strains of Influenza vaccine were significantly higher than those measured before vaccination in each of the three groups. Also, GMTs against Influenza B strain in the Cirrhotic patients was more than inactive carriers of Hepatitis B and healthy subjects (Table 2).

Liver cirrhosis is a major chronic disease in many Asian countries(15), that is mainly due to chronic Hepatitis B(17), and Hepatitis B virus (HBV) infection remaining the main cause of chronic liver disease in Iran.(5,6) Influenza and its complications are known as a disease which increases in patients with underlying chronic medical conditions, such as cirrhosis.(15) In addition, Influenza virus may induces pro-inflammatory cytokines, such as IL-1, IL-6 and TNF-α and causes hepatic injury.(18) Therefore, influenza prevention with vaccination may play an important role in reduction of severe complications of influenza in patients with chronic liver disease.

In this study, the immunogenicity of Influenza vaccination was evaluated in patients with cirrhosis and inactive carriers of Hepatitis B. The Seroconversion rate in Cirrhotic patients against the three vaccine strains ranged from 71.4% to 100% which was indicative of the vaccine’s capability to induce immunity in this group. The antibody titer measured by use of GMTs before and after vaccination is suggestive of significant antibody production in all the groups. Also, Seroconversion rate and antibody GMTs against H3N2 and H1N1 strains for Cirrhotic patients and inactive carriers of Hepatitis B showed no significant difference compared with the healthy subjects. The Seroprotection rate, which is was indicative of the capability of the vaccine in inducing protection against affliction with the disease, was 100% in Cirrhotic patients against all strains. This rate in inactive carriers of Hepatitis B was ranged from 91.20% to 100% and in the healthy subjects from 93.6% to 96.8% and showed no significant statistical difference between the three groups. These results suggested the excellent immunogenicity of Influenza vaccination in Cirrhotic patients as well as in inactive carriers of Hepatitis B and healthy subjects.

Interestingly, the Seroconversion rate and antibody GMTs against B strain in the Cirrhotic patients were remarkably more than what was in the inactive carriers of Hepatitis B and the healthy subjects. This finding may only shows the effective immunogenicity of B component of Influenza vaccine in Cirrhotic patients. Although γ globulins which is produced by B lymphocytes are increased in cirrhosis due to increased synthesis of antibodies, some of which are directed against intestinal bacteria that liver fails to clear through the hepatic circulation19, there is no evidence that Cirrhotic patients have better Humoral Immune response than healthy subjects. It seems that this finding is probably due to some important epidemiologic consideration. As it’s shown in Table 2, baseline Sero-protective levels and antibody GMTs against B component of Influenza virus in Cirrhotic patients was significantly higher than inactive carriers of Hepatitis B and healthy subjects. It may be due to higher mean age of these patients and more previous exposure to similar B strains of Influenza virus. We know that significant differences are found in epidemiology, genetic organization and antigenic variation between the Influenza virus types. In Influenza virus type A, in addition to minor antigenic drift, antigenic shift can leads to major variation in Influenz virus and produces new viruses to which the population has no immunity. There is a little/no serologic relationship between the antigens of the old and new viruses, whereas Influenza virus type B has only antigenic drift that refers to relatively minor antigenic changes in subtypes. A variable degree of cross-protection within a subtype has been observed. Therefore more previous exposure to similar B strains of Influenza virus may leads to more production of protective antibody in older persons. Moreover, memory T-lymphocyte responses may play a role in ameliorating the severity of disease and speeding recovery after infection in persons with more previous exposure to similar strains of Influenza virus.(7) This may explains why Cirrhotic patients in this study had significantly more Seroconversion rate and antibody GMTs against B strain, after vaccination, than that in the inactive carriers of Hepatitis B and the healthy subjects.

In two similar surveys were performed by Gaeta and his colleagues in 2002 and 2009, the rate in Cirrhotic patients was reported 75% to 85% and 42.1% to 91.3%, respectively. In both surveys, the Seroconversion and Seroprotection rates in Cirrhotic patients showed no substantial differences compared to healthy subjects. Indeed, antibody production after vaccination against the three vaccine strains was high in cCirrhotic patients with no difference compared to the healthy subjects(12,13).

In the present study, three indices, i.e. Seroprotection, seroconversion and antibody GMTs, were used to estimate the immunogenicity of Influenza vaccine in the subjects. As it was shown, all three indices confirmed convenient response in Cirrhotic patients and inactive carriers of Hepatitis B against Influenza vaccine, which showed good immunogenicity compared to healthy subjects.

Influenza mortality is higher in patients with underlying chronic diseases. Here, Cirrhosis is one of the main chronic diseases in many countries.(14,15) Based on the World Health Organization (WHO) report, chronic liver diseases are the cause of more than 1.4 million mortality cases in a year and one of the 10 ain causes of death worldwide. Immune system dysfunction in Cirrhotic patients is a the product of numerous pathophysiologic mechanisms of which one can mentions the reticuloendothelial dysfunction, defect in hepatic production of complement components, weakening of neutrophils and T and B lymphocytes function abnormal production of Immunoglobulins and macrophage dysfunctions.(10)

However, Influenza can induces the systemic effects in the afflicted patients which leads to aggravation of chronic hepatic diseases of which one can mentions direct damages to Hepatocytes by Influenza virus, damages which are induced by circulating cytokines in the course of Influenza disease, and seriousness of the underlying pulmonary disturbances in Cirrhotic patients.(11) Indeed, in patients with chronic liver disease, Influenza and the secondary bacterial infections which are emanated from it may lead to increased load of severe complications includes admissions to hospital and mortality.(12)

Therefore, it seems that prevention of Influenza in patients with chronic liver disease, particularly in Cirrhotic patients, is a logical measure which can extensively reduces mortality and complications. Despite the fact that the immunogenicity of Influenza vaccine in Cirrhotic patients has been confirmed in the present study and numerous other studies(12,15), but annual Influenza vaccination in Cirrhotic patients is lower than what was expected. In Arguedas et al’s study which was done in the domain of vaccination coverage in Cirrhotic patients, the annual Influenza vaccination coverage was only 55% (20), while in the present study no Cirrhotic patient had been vaccinated against Influenza virus up to the date of investigation (Table 3).

Although ACDC has not enlisted the subjects with chronic hepatic diseases as the patients in need of annual vaccination (16), but according to the details were mentioned before, it seems that annual Influenza vaccination in Cirrhotic patients is necessary.

## Conclusion

Patients with chronic hepatic diseases are vulnerable to the occurrence of Influenza complications and mortality due to being afflicted with immune system disturbances. Influenza vaccination is an efficacious way for prevention of these serious consequences.
